# Enfermar, curar y administrar las dolencias venéreas: ciudadanía sanitaria en Argentina, 1900-1960

**DOI:** 10.1590/S0104-59702025000100035

**Published:** 2025-09-15

**Authors:** Ailin Basilio Fabrisi

**Affiliations:** i Becaria Doctoral, Consejo Nacional de Investigaciones Científicas y Técnicas, Instituto de Investigaciones en Humanidades y Ciencias Sociales/Facultad de Humanidades y Ciencias de la Educación/Universidad Nacional de La Plata. La Plata – Argentina ailinbasiliofabris@gmail.com

Las preocupaciones y las respuestas que distintas sociedades han dado al origen, la propagación, la atención, la cura y la prevención de las enfermedades revelan aspectos de una época y de quienes la viven. Volver la mirada al pasado es saber que no todas gozaron de la misma consideración social, política y moral. De hecho, la reputación de las enfermedades reposa en componentes biológicos y sociales anclados en escenario históricamente cambiantes.

En la Argentina de las primeras décadas del siglo XX, las dolencias venéreas fueron objeto de un tratamiento diferencial por parte de galenos, publicistas, científicos, farmacéuticos, juristas, funcionarios y dolientes. La sífilis, la gonorrea, la blenorragia, entre otras, desvelaron a sectores de distinta extracción ideológica, alarmados por los efectos negativos en la reproducción racial de la población. Dicho discurso se inscribía en un contexto marcado por distintos debates políticos, innovaciones técnicas y transformaciones sociales de alcances y escalas variadas. Esas preocupaciones morales, médicas y políticas coagularon en la sanción y la reglamentación de la ley n.12.331 en 1936.

El libro *Ciudadanía enferma: las venéreas en Argentina, 1900-1960* (Biernat, 2024) analiza los factores que hicieron de las dolencias de transmisión sexual un problema público. Su autora, Carolina Biernat, demuestra hasta qué punto la construcción del Estado y la nación necesitó imaginar, delimitar y concretar representaciones, arreglos y prácticas ligadas al cuerpo sano. ¿En qué medida la salud actúa como credencial y criterio para integrar la comunidad social? ¿Cómo las enfermedades venéreas (los actos de enfermarse y curarse) ponen en juego y exponen ansiedades y temores raciales, de clase y de género? Estos interrogantes responden a una argumentación que evidencia cómo las venéreas sirvieron a la construcción de un proyecto político mayor que homologó el cuerpo sano individual a una nación moderna, pujante y colectiva.

La obra se inscribe en una historia social y cultural de la salud y la enfermedad de extensa trayectoria, y en actual expansión, en Argentina y América Latina (Armus, 2010; Belmartino, 2005). Con suma claridad expositiva, la autora recoge y dialoga con el conjunto de trabajos recientes que observaron la importancia de la salud, el avance de la ciencia y la expansión del mercado (publicidad, medicamentos, consultorios médicos) a lo largo de las primeras décadas del siglo XX (Ortiz Bergia, 2022). El libro comprende a las enfermedades venéreas como vectores de un cambio más amplio ligado a la concepción del individuo y, con él, del Estado y la nación en cuanto a la construcción de una comunidad política sana, responsable y productiva (Simonetto, 2019).

Una de sus principales contribuciones es el concepto “ciudadanía sanitaria”. En él convergen las inclusiones y las exclusiones, las violencias y las negociaciones entre los distintos protagonistas y las instituciones. Dicho concepto funciona como hilo conductor teórico y metodológico para entender y presentar los distintos tópicos que conformaron las políticas sociales en relación con las dolencias venéreas. Para conseguirlo, la autora recurre y analiza fuentes de distinto origen (expedientes judiciales, revistas de interés general, debates parlamentarios, legislación, correo de lectores, publicaciones médicas, ensayos políticos, publicidades, afiches).

*Ciudadanía enferma* hilvana una trama historiográfica en la que resuenan distintos procesos históricos. En sus casi doscientas páginas, se aborda y discute la construcción del Estado en clave institucional y burocrática (las campañas de profilaxis, el diseño de políticas públicas y la acción de los organismos), la expansión del mercado farmacéutico y las estrategias publicitarias, la validación social de la medicina, el afianzamiento del sistema científico, los discursos eugenésicos a cargo de las élites médicas y políticas y la definición de la salud como problema de orden colectivo. La obra se compone de cinco capítulos, una introducción y un epílogo. Cada uno de ellos se organiza alrededor de un aspecto del objeto de investigación: imaginar, curar, prevenir, juzgar y aislar. Esta decisión escapa a los procedimientos habituales basados en criterios cronológicos. El resultado es una argumentación clara y fluida, que articula sólidamente lo teórico y lo empírico y que plantea inferencias críticas respecto de los tiempos y las decisiones de los distintos actores involucrados.

Son especialmente destacables los últimos capítulos por dos motivos. El primero, por subrayar la mirada y las acciones desplegadas por los/as dolientes respecto de las denuncias por contagio y el confinamiento obligatorio previsto por la Ley de Profilaxis Venérea de 1936. Lejos de atribuirles un rol pasivo, los sujetos enfermos entendieron que los juzgados y las salas de hospitales eran escenarios para tensionar, navegar y negociar el saber/poder que los colocaba bajo el foco del estigma y fuera de la ciudadanía sanitaria. La interpretación de registros judiciales, expedientes médicos y revistas ilumina los contactos y las “traducciones”, como señala Biernat, que las personas enfermas hicieron de su propio padecimiento a partir de la imaginación profiláctica de las agencias estatales, de los jueces y de los médicos. El segundo motivo, por observar, señalar y analizar los límites y los alcances del Estado en la implementación de instrumentos legales y punitivos en su pretensión por lograr un grado deseable de ciudadanos integralmente sanos. Los empeños puestos por las reparticiones sanitarias son indicios sugerentes para reflexionar sobre cómo se legitima un Estado a los ojos de sus habitantes y los mecanismos disponibles para lograrlo. La construcción sociocultural de las dolencias venéreas pone de relieve las dislocaciones y las torsiones entre la gestión política, la legislación y los actores.

En suma, *Ciudadanía enferma* es una obra necesaria en tiempos donde se debaten las consecuencias de la pandemia de covid-19 y los casos de sífilis aumentan en los segmentos más jóvenes de la sociedad. La irrupción de un virus en 2020 y las decisiones sanitarias para prevenir, tratar y erradicar las dolencias nos recuerda que en los miedos y las ansiedades contemporáneas resuenan sus homólogos pretéritos.
